# Low Oxygen Response Mechanisms in Green Organisms

**DOI:** 10.3390/ijms14034734

**Published:** 2013-02-27

**Authors:** Valeria Banti, Beatrice Giuntoli, Silvia Gonzali, Elena Loreti, Leonardo Magneschi, Giacomo Novi, Eleonora Paparelli, Sandro Parlanti, Chiara Pucciariello, Antonietta Santaniello, Pierdomenico Perata

**Affiliations:** 1 PlantLab, Institute of Life Sciences, Scuola Superiore Sant’Anna, Via Mariscoglio 34, Pisa 56124, Italy; E-Mails: v.banti@sssup.it (V.B.); b.giuntoli@sssup.it (B.G.); s.gonzali@sssup.it (S.G.); g.novi@sssup.it (G.N.); e.paparelli@sssup.it (E.P.); s.parlanti@sssup.it (S.P.); c.pucciariello@sssup.it (C.P.); a.santaniello@sssup.it (A.S.); 2 Institute of Agricultural Biology and Biotechnology, National Research Council, Via Moruzzi 1, Pisa 56100, Italy; E-Mail: loreti@ibba.cnr.it; 3 Institute of Plant Biochemistry and Biotechnology, University of Münster, Schlossplatz 8, Münster 48143, Germany; E-Mail: magneschi@uni-muenster.de

**Keywords:** anoxia, *Arabidopsis thaliana*, *Chlamydomonas reinhardtii*, hypoxia, low oxygen, *N*-end rule, *Oryza sativa*

## Abstract

Low oxygen stress often occurs during the life of green organisms, mostly due to the environmental conditions affecting oxygen availability. Both plants and algae respond to low oxygen by resetting their metabolism. The shift from mitochondrial respiration to fermentation is the hallmark of anaerobic metabolism in most organisms. This involves a modified carbohydrate metabolism coupled with glycolysis and fermentation. For a coordinated response to low oxygen, plants exploit various molecular mechanisms to sense when oxygen is either absent or in limited amounts. In *Arabidopsis thaliana*, a direct oxygen sensing system has recently been discovered, where a conserved *N*-terminal motif on some ethylene responsive factors (ERFs), targets the fate of the protein under normoxia/hypoxia. In *Oryza sativa*, this same group of ERFs drives physiological and anatomical modifications that vary in relation to the genotype studied. The microalga *Chlamydomonas reinhardtii* responses to low oxygen seem to have evolved independently of higher plants, posing questions on how the fermentative metabolism is modulated. In this review, we summarize the most recent findings related to these topics, highlighting promising developments for the future.

## 1. Plant Metabolic Regulations under Low Oxygen

### 1.1. A Shift towards the Fermentation Pathway

Plants and algae rely on aerobic respiration to produce energy. Oxygen availability is thus required and changes in its accessibility lead to drastic metabolic rearrangements. Ultimately, aerobic organisms die if the absence of oxygen is prolonged. A lower level of oxygen can result from environmental conditions, but also anatomical and tissue constraints. During their life, plants and algae are exposed to a range of oxygen concentrations that can vary from a fully aerobic state (normoxia) to oxygen deficiency (hypoxia) or the total absence of oxygen (anoxia) [1]. Excessive rainfall can lead to soil waterlogging or even to the complete submergence of plants, with dramatic consequences for crops. In the context of climate change, an increased frequency of flooding has been observed worldwide [2]. Under hypoxia or anoxia, a series of rapid and profound molecular and metabolic responses are activated to endure such stress.

Oxygen deprivation compromises ATP production and energy supply. This is because oxygen is the final acceptor of electrons in the mitochondrial oxidative phosphorylation occurring during respiration [3,4]. Under submergence oxygen availability decreases drastically and oxygen production by photosynthesis is reduced by water turbidity. Reduced light availability in plants that are submerged also compromises carbohydrates production by the Calvin cycle. Fermentation is required because the mitochondrial production of ATP is hampered in the absence of oxygen. Under these conditions, plants have to rely on the small amount of ATP that can still be produced through glycolysis. A lack of the mitochondrial re-oxidation of NADH puts glycolysis at risk of stopping soon after the low oxygen conditions have been established, unless an alternative mechanism for NADH re-oxidation is activated, *i.e.*, fermentation [5,6]. In some cases of oxygen absence, the “Pasteur effect” may occur, which accelerates the carbohydrate consumption with a 2 to 3 fold increase in the glycolytic flux with respect to the aerobic control [7]. Under oxygen deficiency, the plant metabolism uses pyruvate as a starting substrate to switch on two main pathways: ethanolic and lactic fermentations [8].

**Figure 1:**
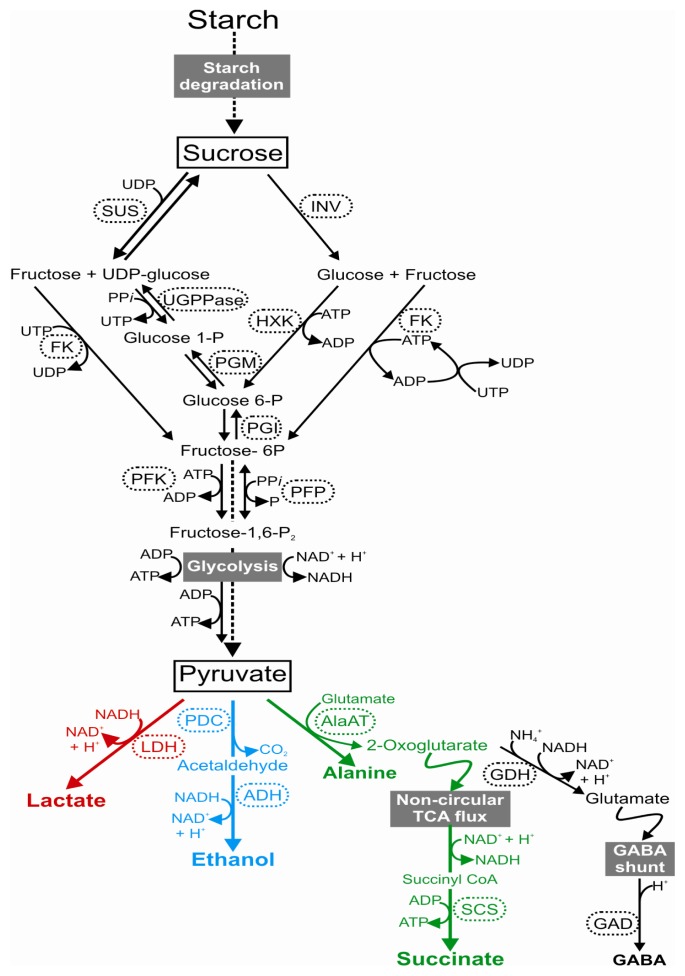
Under low oxygen stress, plants reconfigurate their metabolism to maximize ATP production. Three fermentative pathways are activated using the pyruvate as a starting substrate, deriving from sugar catabolism and glycolysis. The direct products of these pathways and other metabolites involved in this mechanism are indicated in bold. Abbreviations: AlaAT, alanine aminotransferase; ADH, alcohol dehydrogenase; GABA, γ-aminobutyric acid; GAD, glutamic acid decarboxylase; FK, fructokinase; GDH, glutamate dehydrogenase; HXK, hexokinases; INV, invertase; LDH, lactate dehydrogenase; PDC, pyruvate decarboxylase; PFK phosphofructokinase ATP-dependent; PFP, phosphofructokinase PPi-dependent; PGI, glucose-6-phosphate isomerase; PGM, phosphoglucomutase; SCS, succinyl CoA; SUS, sucrose synthase; TCA, tricarboxylic acid; UGPPase, uridine diphosphate glucose pyrophosphatase; UDP-glucose pyrophosphorylase.

In almost all plant species, a rapid activation of lactate dehydrogenase (LDH), which catalyzes the reduction of pyruvate to lactate (Figure 1), has been observed under low oxygen conditions [9–12]. The production of lactate, however, exerts a negative effect on cytoplasmic pH, and therefore the production of lactate is only transient and replaced by ethanolic fermentation. The interaction between lactic and ethanolic fermentation leads to the tight control of the cytosolic pH of cells [13]. In fact, lactic fermentation acidifies cytoplasm in order to obtain an optimal value for PDC activity, with the consequent activation of ethanol production to avoid any deleterious cytoplasmic acidification [13,14]. Studies of several species, such as tomato, potato, Arabidopsis and rice, have shown that lactate production plays a minor role in low oxygen responses, because lactate is generally produced only during the first hours of stress and is easily expelled from the cell, in order to prevent an excessive accumulation of this compound in the cytosol [15]. Some proteins, such as the hypoxia-inducible Nodulin Intrinsic Protein (NIP2; 1) [16], have been indicated as members of the lactate extrusion process. However, an increase in root tolerance to hypoxia has been reported when *LDH* was over-expressed in Arabidopsis plants [17]. This resulted in a significantly higher activity of PDC, suggesting that the lactic and ethanolic fermentation pathways are inter-dependent and that the lactate metabolism is involved in the enhancement of ethanol production [17].

Ethanolic fermentation consists of the conversion of pyruvate to ethanol through the coupled reactions of pyruvate decarboxylase (PDC) and alcohol dehydrogenase (ADH) (Figure 1). The significant role of both ADH and PDC for hypoxia survival has been demonstrated in many plant species, which differ in their degree of low oxygen tolerance, such as maize, rice, *Rumex palustris* and *Arabidopsis thaliana*[18–23]. Although the conversion of pyruvate to acetaldehyde, catalyzed by PDC, is considered to be rate-limiting in ethanol fermentation, the regulatory role of PDC has not yet been fully described. In several plant species, PDC controls anaerobic sugar catabolism and its over-expression in transgenic tobacco leaves has been shown to produce 10 to 20 fold more ethanol than wild-type plants under anoxia [24]. In Arabidopsis, there are four genes encoding PDC, and microarray datasets related to low oxygen conditions have shown that *PDC1* and *PDC2* genes are induced when oxygen deprivation occurs [25,26], whereas *PDC3* and *PDC4* mRNA levels are not markedly induced by anoxia [26].

Intriguingly, the overexpression of *PDC1* and *PDC2* in Arabidopsis plants has been shown to improve ethanol production and survival under low oxygen conditions [27]. Furthermore, it has been reported that *pdc1* null mutants are less tolerant to anoxia, suggesting that the *PDC1* gene is needed in order for the plant to survive the stress [25]. A recent detailed investigation of *pdc1* and *pdc2* mutants also indicated that *PDC1* plays a predominant role in roots, while *PDC2* is leaf-specific [26].

In *ADH* knock-out plants, a general reduction in low oxygen tolerance has been observed [20,27]. The Arabidopsis *adh1* null mutants showed a lower survival rate under hypoxia [28]. In rice, *rad* (reduced adh activity) mutants displayed a reduction in the ADH protein due to a point mutation in the *ADH1* gene, and, consequently, a higher sensitivity to submergence stress (*i.e.*, reduced coleoptile elongation) [29,30]. In maize, the *adh1*^−^ mutant, affected in the biosynthesis of ADH, has been found to be very susceptible to anoxia [31]. Conversely, an increased tolerance to hypoxia, together with an enhanced root growth, has been obtained when *ADH* was over-expressed in Arabidopsis hairy roots [32], but this had no effect on the ability of Arabidopsis transgenic plants to survive flooding [27].

Finally, Ala, γ-aminobutyric acid (GABA) and succinate have been suggested as additional products of the anaerobic metabolism [15,33,34]. Under anaerobic conditions, Ala is accumulated by the transfer of an amino group from glutamate to pyruvate, generating 2-oxoglutarate (2-OG) as a co-product, thus causing a decrease in glutamate content (Figure 1) [35]. This reaction is mediated by the Ala aminotransferase enzyme (AlaAT) and may contribute efficiently to tolerance, although how is still unknown, since Ala accumulation is not toxic for the cells [36,37]. Upon anoxia, the 2-OG can be metabolized via two different shunts [2,15]. In fact, 2-OG can again be oxidized by glutamate dehydrogenase (GDH), with the incorporation of NH_4_^+^ and the generation of NAD^+^, to glutamate, which is then decarboxylated to GABA by glutamate decarboxylase (GAD), a process that is stimulated in acidic conditions. The production of GABA and the next transamination to succinic semialdehyde may help to stabilize the cytosolic pH [38] and ensure an additional conversion of pyruvate to Ala [39]. Alternatively, 2-OG can be used as a substrate in the non-circular TCA flux and thereafter metabolized to succinate via the succinyl CoA ligase enzyme (SCS) [34,40]. To fuel this process, NADH is oxidized by the reduction of oxaloacetate by malate dehydrogenase (MDH) to malate, which is further converted to fumarate and succinate.

Overall, this evidence has shown that both ADH and PDC play a key role in low oxygen tolerance. Other products of the anaerobic metabolism can increase plant tolerance, however their regulation is still poorly understood and needs investigating further. For decades, plants metabolic mechanisms have been studied because of their significance in low oxygen tolerance, and it is clear that this will remain an exciting field of study. In the future, it will be important to deepen the current knowledge on the transcriptional regulation of metabolic genes during low oxygen responses, also in relation to the recent identification of the oxygen-sensing mechanism that seems to be at the basis of ethanolic fermentation activation [41,42] (see section 2.1), which represents a challenging starting point.

### 1.2. Sugar Metabolism under Low Oxygen

Sugars metabolism can be regulated in response to many environmental signals, which directly or indirectly influence the synthesis, concentration, transport and storage of sugars [43,44]. Under low oxygen conditions, the shift from aerobic to fermentative metabolism requires constant carbohydrate supplementation. Indeed, maintaining adequate levels of readily fermentable sugars in hypoxic or anoxic roots is one of the key adaptive mechanisms to an oxygen-deficient environment [45,46]. Several studies have found a close association between carbohydrate utilization and tolerance to low oxygen conditions [47–49]. Anoxia or submergence tolerance has in most cases been associated with a slower use of carbohydrates, rather than with their relative abundance. Studies of the mechanism behind the tolerance of riparian plant species, such as *Salix variegata*, *Arundinella anomala* and *Althernanthera philoxeroides*, have revealed that the carbohydrate metabolism plays a very important role [46]. The ability to survive a long-term oxygen deficiency was explained by the capacity of these riparian species to preserve carbohydrate reserves, in contrast to the large decrease in carbohydrate concentrations observed in intolerant species [50]. The maintenance of adequate carbohydrate availability after prolonged anoxia or submergence is considered a direct consequence of a well-modulated fermentative metabolic activity, which is a strategy of some tolerant species [51]. In storage organs such as grass grains, potato tubers and *Acorus calamus* rhizomes, the supply of hexoses for glycolysis is guaranteed through an efficient starch mobilization via starch-degrading enzymes, such as endo-amylases, exo-amylases, debranching enzymes and starch phosphorylase [18,33,52,53]. Exogenous sugars supply can affect the ability to survive under low oxygen. The supplementation of sucrose, but not glucose, improves survival under anoxia [23,54]. In addition, the role played by sucrose in affecting anoxia responses has been investigated in terms of the transcriptome profile, revealing that this disaccharide modulates the anoxia-driven induction of a plethora of genes, different from the typical anaerobic ones as well. In order to provide hexose 6 phosphate for glycolysis, sucrose degradation takes place through two distinct pathways in plant cells: the sucrose synthase (SUS) bi-directional pathway, favoured in the catabolic direction, and the unidirectional invertase (INV) pathway (Figure 1). Furthermore, SUS (rather than INV) is reported to be the preferential pathway for sucrose degradation when oxygen is limited [55]. The SUS pathway is favoured under low oxygen because it partially preserves the energy of the glucose-fructose bound. While the INV pathway cleaves the sucrose molecule into glucose and fructose, the SUS reaction releases fructose and UDP-glucose, which can be subsequently utilized by UDP-glucose pyrophosphorylase (UGPPase), producing UTP and glucose-1P. UTP is then utilized by fructokinase (FK). The conversion of sucrose into phosphorylated hexoses, does not therefore require ATP, whereas the INV pathway requires 2 mol of ATP per mole of sucrose [56,57]. Studies on maize roots have shown that both sucrose synthase genes *SUS1* and Sh1 were upregulated by low oxygen [58]. In Arabidopsis, *SUS1* and *SUS4* transcripts, as well as their relative proteins, increase significantly upon oxygen deprivation both in roots and shoots [59]. This leads to the hypothesis that SUS plays a central role in the sucrose metabolism under conditions of oxygen deprivation, contributing to the degradation of the disaccharides, and thus to the availability of substrates for energy production through glycolysis and fermentation. Indeed, Bieniawska and colleagues [60] showed that double mutant plants (*sus1* × *sus4*) are less tolerant to 4-day root submergence compared to wild types.

Glucose and fructose need to be phosphorylated by hexokinases (HXK) to be channeled to the glycolytic pathway. In rice seedlings, the induction of the fructokinase *OsFK2* has been shown, together with the hexokinase *OsHXK7*[61], to be likely induced via hypoxia-driven sucrose starvation [62,63].

Other glycolytic reactions may utilize the PPi available instead of ATP under oxygen deprivation, improving the net yield of ATP per mol of sucrose catabolized. The phosphorylation of fructose-6P to fructose-1,6P2 can be catalyzed by the unidirectional phosphofructokinase (PFK), which uses ATP as a phosphate group donor, or via the bidirectional PFK-PPi (PFP) which uses PPi. PFP is activated under anoxia in rice [64,65] and, together with PDC, it can regulate the carbon flux during anaerobiosis in different plant species [7,65,66]. Since the PFK step can be limiting under oxygen deprivation, the PPi supply may be increased by a substrate cycle coupled with pyruvate kinase (PK) and pyruvate orthophosphate dikinase (PPDK), in order to finally accelerate glycolysis. Interestingly, PPDK is up-regulated by low oxygen in rice, both at the gene and protein levels [63,67].

Many studies suggest that submergence tolerance in several plant species may be associated with the ability to mobilize carbohydrate reserves, and also that the most important enzymes, which are involved in the degradation of carbohydrates, seem to play a very important role.

## 2. Low Oxygen Sensing and Signaling in *Arabidopsis thaliana*: The *N*-End Rule Pathway (NERP) and Side Defense Mechanisms

### 2.1. Oxygen Sensing in Plants

Metabolic alterations under low oxygen might in theory be exploited by plant cells to sense hypoxia and generate adaptive responses. Indirect oxygen-sensing mechanisms could be based on low energy status, variations in carbohydrate availability, reactive oxygen species (ROS) signals, nitric oxide (NO)-related responses, NAD/NADH ratio, calcium fluxes and homeostatic reactions to intracellular pH changes [15]. However, the oxygen status might also be directly measured by the cell, for example through sensor proteins or transcription factors whose activity is directly affected by oxygen levels. Indeed, direct oxygen-sensing systems have been described for metazoans, yeasts and microorganisms. In all animal taxa studied so far, the perception of hypoxia relies on the stabilization of the heterodimeric factor HIF, a protein belonging to the bHLH-PAS domain family of eukaryotic transcription factors, which has been recognized as the unique activator of the whole set of hypoxia-inducible genes. In fact low oxygen prevents prolyl hydroxylation of the animal HIFα subunit, which under normoxic conditions leads to the ubiquitination and consequent degradation of the protein [68,69]. Plant genomes encode both PAS domain proteins and prolyl hydroxylases (PHDs), some of which are low oxygen and/or redox regulated. Therefore, the presence of HIF-like signal transduction pathways has also been hypothesized in plants.

However, it has been suggested that plant PAS and PHD proteins have functionally diverged from animal proteins, acquiring exclusive roles in the transduction of light signals and the modification of the cell wall, respectively [70]. In algal cells, proline hydroxylation occurs in cell wall proteins, and several PHDs have been found to be induced in *Chlamydomonas* under anaerobiosis [71]. However, it is unlikely that this process occurs under anoxia, since it requires oxygen as a substrate [71]. Thus, the existence of on oxygen-dependent post-translational modulation of pivotal TFs needs further investigation. This confirmation has been obtained recently in plants, when two independent studies showed that oxygen availability directly impacts on the stability of the transcriptional activator RAP2.12, through the *N*-End Rule Pathway (NERP) for protein degradation [41,42].

NERP is an ubiquitin-dependent proteolytic system that links the half life of a peptide to its *N*-terminal amino acidic sequence [72]. *N*-terminal residues can be classified as stabilizing or destabilizing, depending on their ability to prevent or promote, respectively, the recognition of the protein by the components of the proteolytic machinery. In eukaryotic cells, amino-terminal degradation signals (*N*-degrons), corresponding to *N*-terminal destabilizing residues, can be specifically bound by E3 Ubiquitin (Ub) ligases, therefore called *N*-recognins, which, after recruiting Ub-activating (E1) and Ub-conjugating (E2) enzymes, target short-lived proteins to the 26S proteasome for degradation [73]. Structural determinants for a functional *N*-degron include an *N*-terminal destabilizing residue, a neighbouring Lys residue for ubiquitin conjugation, and the vicinity of a flexible region facilitating its exposure [72,74]. An *N*-degron can be created through the endoproteolytic cleavage of proteins, the removal of the *N*-terminal signal or transit peptides, or the elimination of NH_2_-Met [73,75].

In both eukaryotic and prokaryotic organisms, the NERP displays a common hierarchical organization, in which primary N-terminal destabilizing residues, directly bound by the *N*-recognins, can be produced by a sequential modification of secondary or tertiary destabilizing residues [76]. Plants and mammals share identical sets of destabilizing residues [77]. Primary residues consist of basic (Arg, Lys and His, “type I” residues) or bulky hydrophobic amino acids (Phe, Trp, Tyr, Leu and Ile, “type II” residues). Secondary residues are Asp, Glu or oxidized Cys, all of which, in the *N*-arginylation branch of the NERP, are substrates for the conjugation of Arg by Arg-tRNA transferase (ATE) enzymes. Secondary residues can be generated by *N*-terminal amidohydrolases (Nt^N^- and Nt^Q^-amidases), through the deamidation of Asn and Gln, or by oxidation of Cys, which therefore act as tertiary destabilizing residues. Proteins are generally synthesized with an *N*-terminal stabilizing Met residue, which however can be removed by the action of Met-aminopeptidase enzymes (MetAPs), provided that the following amino acid is characterized by a short side chain. Of such residues, Cys is the only one whose exposure can further lead to the creation of an eukaryotic *N*-degron [78].

After the disclosure of the NERP mechanism and the acknowledgment of its conserved organization across evolution, enzymatic players in plants were rapidly identified by forward genetic and sequence similarities, revealing a certain degree of divergence from the animal kingdom [79]. However, the physiological significance of the pathway in plants has started to be elucidated only recently and is mainly derived from the genetic analysis of components of the *N*-arginylation branch, which targets only a subset of all possible substrates to degradation. The Arabidopsis ATE1, which was first reported to be involved in leaf senescence [80], has also been demonstrated to work redundantly with its homolog ATE2 in rosette development and leaf shape determination [81]. Of the two plant *N*-recognins identified to date, named PRT1 and PRT6, only the latter is associated with a recognizable mutant phenotype in Arabidopsis, mainly characterized by a stronger seed after-ripening dormancy, which was found to correlate with hypersensitivity to ABA [82].

The finding that plant oxygen sensing relies on the *N*-terminal mechanism for protein destabilization thus provides the first evidence of the involvement of the NERP in plant stress responses. A conserved *N*-degron, whose consensus sequence is MCGGAI/L, was discovered in group VII ERF transcription factor proteins, and recognized as the key for the group VII ERF-driven activation of the downstream transcriptional events that are the signature of plant anaerobic responses. Members of this subfamily of the plant-specific ERF family [83], such as Arabidopsis Hypoxia Responsive ERF (HREs) and RAP2.2/2.12 and rice SUB1 and SK factors, have long been related to plant transcriptional regulation under low oxygen [84–87] and postulated to work as oxygen-modulated transcriptional switches for acclimation responses.

**Figure 2:**
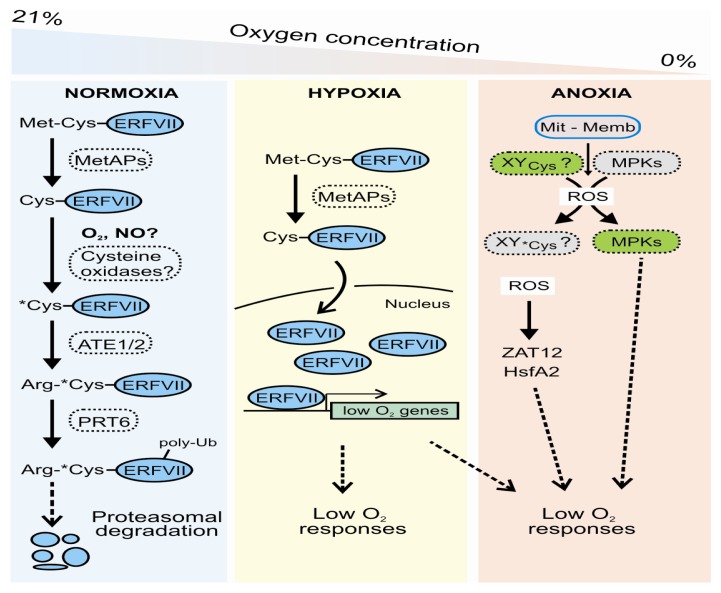
Low oxygen sensing mechanisms in plants are mediated by the *N*-end rule pathway and ROS. Group VII ERF transcription factors bear a conserved Met-Cys *N*-degron that, under normoxia, targets them to constitutive proteasomal degradation, through a branch of the *N*-end rule pathway that relies on the production of oxydized cysteine (*Cys) as an intermediate. Oxygen limiting conditions lead, instead, to stabilization of the Met-Cys *N*-degron, thereby allowing ERFVII to migrate into the nucleus and activate transcription of low oxygen-responsive genes. Low oxygen responses under anoxia also depend on the accumulation of reactive oxygen species (ROS), which triggers the accumulation of ROS-responsive transcription factors and may regulate cysteine-containing enzymes, contributing to the activation of MPKs. This triggers a signaling cascade leading to enhanced plant tolerance. Abbreviations: MetAPs, Met-aminopeptidases; ATE1/2, Arg-tRNA transferase 1/2; PRT6, Proteolysis 6; poly-Ub, poly-ubiquitin chain; MPKs, MAP kinases.

In Arabidopsis, newly synthesized RAP2.12 proteins undergo a constitutive cleavage of the conserved NH_2_-Met-Cys sequence by MetAPs, which leads to exposure of a tertiary *N*-terminal destabilizing Cys residue. In the presence of oxygen, the Cys2 residue can be oxidized to Cys-sulfinic acid (CysO_2_H) or further to Cys-sulfonic acid (CysO_3_H) to trigger the conjugation of the primary destabilizing residue Arg by ATE1/2, the binding of the PRT6 *N*-recognin and the possible targeting of the protein to the proteasome (Figure 2). Under low oxygen conditions, on the other hand, the impairment of Cys oxidation directly translates into RAP2.12 stabilization, thereby allowing the TF to promote the transcription of low oxygen-responsive genes (Figure 2). In line with this, transcriptome analysis of the *ate1ate2* and *prt6* mutants showed a higher level of basal expression of hypoxia marker genes, such as *ADH1*, *SUS4* and *PDC1*[41].

Constitutive degradation of RAP2.12 under normoxia can be prevented, provided that its *N*-terminus is protected from enzymatic modifications. The use of a RAP2.12:GFP fusion construct enabled Licausi and colleagues [42] to observe the sequestration of the protein at the plasma membrane in normal conditions, which turned out to depend on the ability of RAP2.12 to interact with the transmembrane acyl-CoA-binding proteins ACBP1/2. Constitutive availability of “inactive” RAP2.12 bound to the cell membrane is likely to be crucial in making RAP2.12 a true molecular switch for the early response to hypoxia. This is supported by the observation of immediate RAP2.12:GFP displacement from the plasma membrane to the nucleus at the onset of low oxygen conditions. Interestingly, despite all being NERP substrates and sharing overlapping sets of gene targets, RAP2.12 and HRE1/2 factors might diverge functionally. While RAP2.12 mediates the quick activation of the low oxygen response, HRE1/2 could be involved in the long-term acclimation of plants to prolonged oxygen deprivation, as it is accumulated during hypoxia and not constitutively present like RAP2.12 is [42].

It can thus be speculated that the oxygen-dependent NERP mechanism for RAP2.12 turnover is very significant both in preventing mislocalized cytosolic RAP2.12 from reaching the nucleus under aerobic conditions and, more importantly, in driving the degradation of the TF upon reoxygenation of the cell. The restoration of normoxia was consistently followed by the rapid and complete disappearance of the RAP2.12:GFP signal. It is still unknown whether Cys oxidation is spontaneous or enzymatic, however it has been reported to require previous Cys nitrosylation by NO in order to take place [88]. Some cross-talk between oxygen-dependent NERP and NO in oxygen-deprived plant cells is not unlikely, given the known role of NO during hypoxia (see section 2.2).

The importance of the *N*-end rule for plant physiological responses to low oxygen is corroborated by the observation that mutants for the Cys2-dependent NERP branch display altered tolerance to low oxygen stresses. Arabidopsis *ate1/2* and *prt6* mutants, in fact, survived submergence better than wild type plants [41]. However, acclimation of true submergence tolerant species, such as rice, is likely to involve divergence in group VII ERF regulation, as proved by the escape of the SUB1A protein from the NERP regulation (see section 3.1).

### 2.2. A Role for Reactive Oxygen and Nitrogen Species (ROS/NOS)

One of the most controversial aspects related to the molecular response to low oxygen is the presence of oxidative stress. This may seem counterintuitive since oxygen is needed for the production of ROS. However, many different microarray analyses carried out under different low oxygen conditions and on different plants have reported the activation of ROS-regulated genes or those associated with ROS.

*Heat Shock Proteins* (*HSP*s) are the most recurrent of ROS-related proteins that are also induced by anoxia [89,90]. *HSPs* expression has been shown to be associated with the presence of hydrogen peroxide (H_2_O_2_) in different kingdoms [91]. HSPs production has been observed predominantly under anoxia [90], probably as a mechanism needed to activate a defense that overlaps with heat stress [92] (see section 2.3). Some TFs associated with ROS production have also been shown to be regulated by low oxygen, *i.e.*, *HsfA2* and *ZAT12*[90]. Results from yeast and mammalian cells have suggested heat shock transcription factors (*HSF*s) as sensors of H_2_O_2_[93], and *ZAT12* has been shown to be regulated by several stresses where the oxidative signal seems to be involved [94]. These genes have a lower induction in the *rbohD* Arabidopsis mutant under low oxygen stress, suggesting a possible role for this membrane NADPH oxidase superoxide (O_2_^−^) producing enzyme in the mechanism of ROS generation [90].

The ROS produced by the membrane NADPH oxidase was proposed as being part of low oxygen signaling through a ROP rheostat, regulating ADH expression and activity [95]. A more recent report, on the other hand, suggested mitochondria imbalance to be the ROS generator mechanism under oxygen deprivation, in a system supporting survival mediated by mitogen-activated protein kinases (MPKs) activation [96]. Whatever the mechanism, it seems to be transcriptionally independent of the NERP, since under control conditions Arabidopsis mutants associated with this pathway activate anaerobic but not ROS-related genes [90]. An oxidative burst probably orchestrates an alternate pathway of response which, together with the NERP, contributes to tolerance [97]. This is also supported by the finding that plants over-expressing *HsfA2* tolerate low oxygen stress better by activating heat-stress related genes but not the fermentation pathway [92].

One of the most interesting aspects of ROS production under low oxygen is the possible indirect interaction with the NERP. Indeed, this mechanism includes, after an initial Met cleavage at the *N*-end of target proteins, the oxidation of Cys, through a mechanism which may or may not be enzymatic [98] (see section 2.1). Interestingly, Cys residues are also considered to be targets for ROS, and increasing evidence suggests the pivotal role of Cys in ROS perception and network signaling.

Thiol moieties (–SH) on the side chain of Cys are very sensitive to oxidation and can form disulfide bonds in a reversible way, changing the protein state. In parallel, thiol moiety (–SH) on the side chain of Cys can also be subsequently oxidized to a sulfonyl moiety (–SO_3_H), which cannot be reduced under normal intracellular environmental conditions [99]. Indeed, the Cys position in a protein is an important prerequisite for its reactivity towards oxidant or reducing agents, since most thiol groups are either structurally not accessible to redox agents, or the microenvironment is strongly buffered by antioxidants. The Cys pK_a_ value is strictly related to the Cys position, and is a good indicator to establish how prone it is to redox modification. The Cys as thiolate anion (Cys-S^−^) is more readily oxidized than in its thiol form -SH. Since in non-stress conditions the majority of the Cys groups are in a thiol state, few proteins would be expected to be readily susceptible to oxidation. However, Cys may exist as thiolate anions at neutral pH values. Protein Tyr phosphatases (PTP) are examples of proteins with low p*K*_a_ Cys residues (p*K*_a_ = 4.7/5.4), thus reactive as Cys-S^−^ at neutral pH and sensitive to oxidation by various oxidants, including H_2_O_2_[100]. PTP Tyr (de) phosphorylation is involved in the regulation of MPKs activities in plant, fungi and animal cells [101]. Gupta and colleagues [101] showed that *At*PTP1 phosphatase activity is inhibited by H_2_O_2_*in vitro*, suggesting that H_2_O_2_ induces a rapid and reversible Cys-dependent conformation change that leads to inactivation, as demonstrated *in vivo* in animals [102]. Interestingly, H_2_O_2_ treatments have been shown to activate MPKs in plant cells, known to be negatively regulated by PTPs. In particular, *At*PTP1 inactivation has been shown to be correlated to *At*MPK6 activation by H_2_O_2_. In this context, transient *At*MPK6 activation, probably due to ROS of mitochondrial origin, has been observed in response to oxygen deprivation and re-oxygenation and is associated with improved plant survival [96].

The role of NO as a signaling molecule under low oxygen conditions is very promising. NO has been found to be required in the oxidation of the *N*-terminal Cys before arginylation by ATE in mammalian cells *in vivo*[88]. In addition, under low oxygen conditions, nitrite (NO_2_^−^) acts as an alternative electron acceptor and its reduction to NO, conversion to nitrate (NO_3_^−^) by the non-symbiotic hemoglobin Hb1, and then reduction to NO_2_^−^ by nitrate reductase gives rise to a cycle that produces a certain amount of ATP [103,104]. Moreover, recent studies on animals have demonstrated that HIF stabilization and transcriptional activity is achieved also through *S*-nitrosylation of HIF pathway components [105,106].

Since the NERP regulates several different processes in plants other than low oxygen, such as growth and development [98], one interesting hypothesis is that the mechanisms behind Cys stability, and thus the fate of the protein, may be different depending on the situation, either directly regulated by oxygen availability or cellular changes in cytosolic pH or ROS and reactive nitrogen species (NOS) balance. Each of these actors could be involved in the regulation of NERP side mechanisms which, all passing through the modification of Cys located in different positions, could lead to the activation of different downstream events and thus contribute to stress tolerance (Figure 2).

### 2.3. Low Oxygen and Heat Stress Convergence

An intimate overlapping in the activation of a subgroup of *Heat Shock Genes* (*HSG*s) has been observed when comparing the transcriptomes of *Arabidopsis* seedlings under heat, anoxia and heat followed by anoxia, [23,92,107]. A high portion of these genes are strictly related to heat shock (*HSP*s) and oxidative stress-related proteins (*ZAT12* and *AOX1a*) [92,108–110]. A mild heat pre-treatment, able to induce thermotolerance in plants when subsequently exposed to lethal temperatures [111], can also confer a higher tolerance to subsequent anoxia [23,92]. This suggests that two apparently unrelated stress conditions share a mechanism for tolerance.

Of the heat-related genes up-regulated by both heat and anoxia, the heat shock transcription factor *HsfA2* is one of the most strongly modulated [92]. *HsfA2* is involved in several abiotic stress conditions [112–114] and its molecular mechanism of action has recently been described in Arabidopsis [113,115–117]. This transcription factor is assumed to cooperate with other factors to activate the downstream signaling pathway both during the stress period and the recovery phase [113,116,117], and is believed to coordinate the ROS-mediated cross-talk among cellular compartments and organelles [113,117,118]. In addition, a possible direct or indirect involvement of this TF in intracellular pH homeostasis cannot be ruled out, since one of its putative target genes (*ROF2*) plays a pivotal role in this mechanism [119]. Interestingly, *HsfA2* confers increased tolerance to anoxia when over-expressed in Arabidopsis [92]. *HsfA2* over-expression has a positive effect on the expression of small *HSP*s, such as *HSP25.3-P* and *HSP18.2-CI*, and also antioxidant genes, such as *APX2*, *GolS1* and *GolS2*[92,112–114]. *HsfA2* does not affect the expression of any anaerobic genes, suggesting that *HsfA2* does not control the anaerobic pathway [92]. Conversely, the anoxia-driven up-regulation of heat-related genes is independent of the NERP-dependent mechanism of oxygen sensing [41,42]. This suggests that activation of heat-related genes by an anoxic treatment is driven by an independent sensing mechanism that may rely on a ROS-dependent sensing mechanism [90,92,95,120]. Heat-induced tolerance to anoxia is possibly a consequence of the induction of HSPs as well as some antioxidants and ROS-related genes (*ZAT12*, *AOX1a*, *APX2*). These genes may be required during the post-anoxic recovery phase, to protect cells during reoxygenation, which is an oxidative-prone event [121,122].

## 3. Low Oxygen Tolerance Mechanisms in *Oryza sativa*

### 3.1. Rice Germination under Low Oxygen

In terms of tolerance to low oxygen, rice is considered one of the most interesting species. Unlike the majority of higher plants, it is able to germinate under submergence using the starchy reserves of the seeds to fuel the anaerobic metabolism [33]. During early seed germination under submergence, rice growth is restricted to the coleoptile, with both root and primary leaf elongation inhibited [123]. Rice coleoptile elongation under low oxygen enables successful crop establishment in submerged fields, since the long coleoptile can reach the water surface and thus make contact with the normoxic atmosphere [124].

Rice is able to use starch under low oxygen because of the presence of specific enzymes even under anoxia. Of these, α-amylases have a major role in degrading native starch granules [125–127] and catalyzing starch hydrolysis to sugars, which are subsequently translocated to the embryonic axis to fuel glycolysis in order to produce energy for growth. These enzymes are not present in cereals, which are unable to germinate under anoxia or submergence [18,128,129]. Among the various rice isoforms of α-amylases, *Ramy3D* plays a major role for low oxygen survival since it is highly induced under low oxygen [63,130] and does not require gibberellins to be induced [131,132]. Indeed, the synthesis of gibberellins requires oxygen, and the GA-dependent pathway for α-amylase induction is inactive under low oxygen. *Ramy3D* is regulated by a sugar starvation-dependent pathway [133–135]. There is a link between the reduction in soluble sugar content and low oxygen conditions [132–136]. The regulation of *Ramy3D* requires the Calcineurin B-like Interacting Protein Kinase 15 (CIPK15) and the Snf1-Related protein Kinase 1A (SnRK1A), thereby activating the transcriptional regulator MYBS1 that upregulates *Ramy3D*[135,137–139]. A rice mutant defective in CIPK15 (*cipk15*) hardly germinates at all when submerged [135].

The growth of the rice coleoptile under submergence is due to cell expansion, in which expansins are likely to play a role [140,141]. Although the length of coleoptiles under low oxygen does not correlate with expansin expression levels [142], *EXPA7* and *EXPB12* are strongly upregulated in the submerged rice coleoptile and represent good candidates as players in anoxic coleoptile growth [63]. Additionally, the altered expression of *EXPA4* affects coleoptile elongation under submergence in the *japonica* variety Taipei 109 [143].

### 3.2. Strategies to Survive Low Oxygen in Adult Rice Plants

Adult rice plants display an array of adaptive mechanisms for cultivation in a large range of hydrological regimes, from deepwater to flash flooding-prone areas. A considerable variation in submergence tolerance exists among rice genotypes.

The wide number of ecological situations in which rice varieties, landraces and wild genotypes have been grown for centuries, enabled submergence tolerance characters to be selected that are effective in specific habitats. There are two main strategies to enable rice to survive different flooding regimes [84,86] and are also found in distinct wild progenitors [86,144,145]. One of these mechanisms was originally identified in Flooding Resistant 13A (FR13A) [146] a lowland rice landrace able to tolerate several weeks of flooding [147]. Molecular mapping revealed that tolerance was correlated with the *SUBMERGENCE1* (*SUB1*) QTL, located on chromosome 9 [148]. This locus contains up to three ERF genes, including *SUB1A*, which in its allelic form *SUB1A-1*, was recently demonstrated to be the master regulator of submergence tolerance [84]. This gene is activated by ethylene [149]. The near isogenic line M202(*SUB1*) obtained through the introgression of the *SUB1* locus from the tolerant FR13A into the intolerant background M202, revealed that the *SUB1A-1* allele induces the fermentative metabolism under submergence [149] and represses growth through the GA repressors Slender Rice-1 (*SLR1*) and SLR1 Like-1 (*SLRL1*) [150]. SUB1A, therefore, induces a “quiescence” status in the submerged rice plants, enabling them to preserve carbohydrates for use during the recovery phase. *SUB1* marker-assisted backcrossing (MABC) with productive varieties enabled the transfer of the submergence tolerance trait to highly productive mega-varieties [151,152].

Unlike rice varieties that possess the SUB1A gene, deepwater rice (DPR) varieties can elongate very rapidly under submergence. This allows DPR to escape the oxygen-limiting conditions that are associated with flooding by keeping at least the leaf tips above the water surface [86,147]. Stem elongation is driven by two *ERF* genes, named *SNORKEL1* (*SK1*) and *SNORKEL2* (*SK2*), which are driven by ethylene and promote gibberellin-dependent elongation.

*SUB1A-1* and *SK*s belong to the VII ERF group similarly to the Arabidopsis ERFs that are targets of the NERP (*RAP2.2*, *RAP2.12*, *HRE1* and *HRE2*) [41]. However, SUB1A-1 does not seem to be a substrate of the NERP [42], probably because the Met-Cys *N*-degron is made not functional by the absence of a crucial downstream Lys residue or by its structural conformation. In addition, SKs factors harbour variant *N*-termini that deviate from the NERP consensus. The fact that SUB1A-1 and SKs are not targets of the NERP implies that their stability is not affected by oxygen and suggests that these ERFs evolved to take full advantage of ethylene as signaling molecules which accumulate in plants that are submerged [2,97].

## 4. Anaerobic Metabolism in Photosynthetic Microorganisms: The case of *Chlamydomonas reinhardtii*

Photosynthetic microorganisms that inhabit the soil are periodically subjected to hypoxic and anoxic conditions, mainly as a result of day/night fluctuations, high microbial respiration and low rates of oxygen exchange [153]. The photosynthetic soil-dwelling alga *Chlamydomonas reinhardtii* has recently become a good model system for understanding how microalgae adapt to low oxygen levels. In addition to the availability of the nuclear genome sequence [154] and many molecular tools [155–157], knock-out mutants and miRNA-lines have recently opened up new scenarios for green alga [158–161]. Chlamydomonas shares many metabolic features with vascular plants, but it also offers a particular example of eukaryote encoding for proteins usually associated with strict anaerobes such as two oxygen-sensitive [FeFe]-hydrogenase enzymes (HYDA1 and HYDA2, the former being the primarily active hydrogenase isoform [162–165] and the corresponding [FeFe]-hydrogenase maturation proteins (HYDG and HYDEF) [166,167]. Furthermore, pyruvate:ferredoxin oxidoreductase (PFR1), pyruvate formate lyase (PFL1), acetaldehyde/alcohol dehydrogenase (ADH1), phosphoacetyltransferase (PTA1 and PTA2), and acetate kinase (ACK1 and ACK2), typically present in prokaryotes and a few anaerobic eukaryotes [168], are all encoded in the Chlamydomonas genome and involved in the anaerobic metabolism [71,153,169–173].

**Figure 3:**
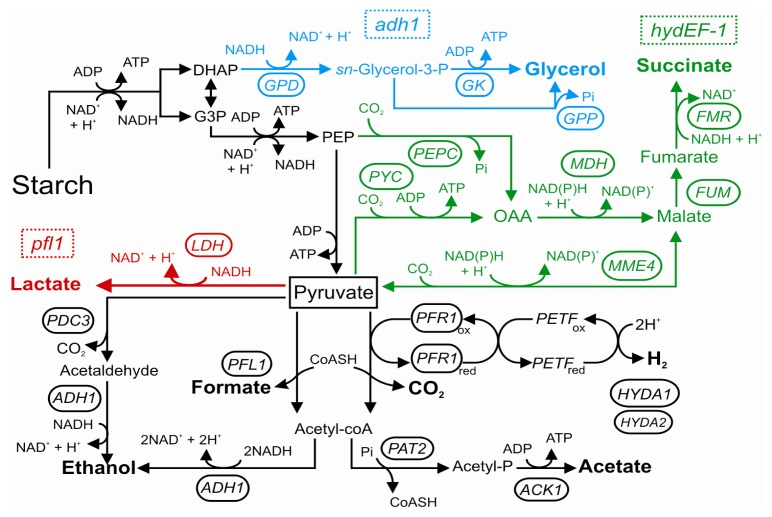
Fermentative pathways of *Chlamydomonas reinhardtii*. Under dark anoxia starch reserves are metabolized to pyruvate through glycolysis, thus generating ATP and reducing power (NADH). Pyruvate is therefore reduced by several fermentative circuits to enable NAD^+^ re-cycling. When specific branches of this fermentation network are impaired (e.g., by artificially knocking out key genes), electron re-routing is observed as highlighted by novel pathways leading to succinate in the *hydEF-1* mutant (green), increased lactate and ethanol in the *pfl1* mutant (red), and glycerol in the *adh1* mutant (light blue). Modified from [153,172,173].

Interestingly, most of these oxygen-sensitive enzymes are located in the chloroplast [153,169,171], suggesting a tight control of fermentation over photosynthetic rates. Activities of these fermentative circuits result in the secretion of organic acids (formate, lactate, malate, acetate and succinate) and alcohols (ethanol and glycerol), with the concomitant evolution of small amounts of CO_2_ and H_2_, the latter being a promising carrier of renewable energy (Figure 3) [71,172–179]. Pyruvate represents the central metabolite of this fermentative network, and differences in the ratio of the fermentative products can occur depending on the culturing conditions and Chlamydomonas strain [71].

Although the precise physiological function of hydrogenase and H_2_ evolution in *Chlamydomonas* has still not been completely resolved, H_2_ production is likely to have evolved as a component of photoprotection functioning as an electron valve for redox poising and fermentative energy production [153]. The metabolic consequences of a lack in functional HYDAs have been reported for the *hydEF-1* mutant strain which does not have the maturation protein necessary for the correct assembly of the HYDA enzymes [179]. In this mutant the reverse TCA cycle (rTCA) is activated to dispose the excess reductants, resulting in high concentrations of extracellular succinate, a metabolite not detected in the parental strain (Figure 3) [179].

Given the extreme sensitivity of the [FeFe]-hydrogenase enzymes, H_2_ evolution in *Chlamydomonas* can only occur when cells are in micro-oxic or anoxic conditions (*i.e.*, during fermentation in the dark or when cells are deprived of sulfur in the light) [180].

The disruption of specific fermentative branches has been suggested as a promising approach for rerouting the fermentative electron flow towards the PFR1-dependent H_2_ production pathway under dark anoxia. Recently, the effects of lesions in PFL1 [170,172] and ADH1 [173] enzymes have been reported. In spite of contradictory results in terms of H_2_ evolution, down-regulation of *in vitro* hydrogenase activity and lower HYDAs and PFR1 transcripts and protein levels in *pfl1* mutants suggest that the H_2_ producing pathway cannot compensate for a loss of PFL1 activity. Instead of competing for the same substrate, PFL1 and PFR1 may actually work together to satisfy the physiological needs of the cell. ADH1 is the only enzyme responsible for ethanol production under dark anoxia in *Chlamydomonas*[173]. Intriguingly, the *adh1* mutant showed no increase in H_2_ evolution, but high levels of extracellular and intracellular glycerol. Synthesis of glycerol at the level of dihydroxyacetonphosphate (DHAP) would allow for NAD^+^ recycling in the mutant strain, thus maintaining viability (Figure 3) [173]. Notably, the disruption of cyclic photosynthetic electron transfer (CEF)—which is induced under low oxygen in the alga—by the deletion of PGRL1 leads to a significant increase in H_2_ production [181]. The manipulation of electron transfer decisions made at the reducing side of photosystem I is therefore a valid instrument to enhance H_2_ evolution from the alga. Moreover, changes in the efficiency of CEF interfere with the overall induction of the anaerobic response in *Chlamydomonas*[182].

The metabolic restructuring observed for insertional mutants suggests that perturbation of the anaerobic metabolism activates unknown pathways that were set in place to regenerate reduced pyridine nucleotides and sustain survival. Additional work aimed at understanding how *Chlamydomonas* readjusts its metabolism to cope with blocks in specific reactions is clearly needed. Indeed, little is known about how photosynthetic microorganisms sense oxic conditions and induce transcription of their fermentative genes. Possible candidates have been proposed, such as the reduction state of the plastoquinone (PQ) pool [183] and the production and detoxification of ROS [184–186]. Prolyl 4-hydroxylases, which directly sense oxygen and are involved in controlling responses to anoxia in animals [184], are also highly up regulated by anoxia in *Chlamydomonas*[71]; their putative role in the green alga, however, is still unknown.

Several fermentative genes follow precise day-night fluctuations in their expression levels, which are not necessarily linked to the photosynthetic oxygen dissolved in the medium [187]. Interestingly, the accumulation of *ADH1* and *HYDA2* transcripts has been shown to be independent of oxygen levels but is instead regulated by the circadian clock and/or cell cycles. *PFR1* and *HYDA1* expressions, on the other hand, are strongly influenced by oxygen tensions [187,188]. These observations raise the question of how and why different branches of the fermentative metabolism are differentially regulated in *Chlamydomonas*.

A comparative transcriptomic reconfiguration study by low oxygen stress suggests the independent evolution of the regulatory networks between *Chlamydomonas* and higher plants [89]. While in general no specific oxygen-responsive transcription factors are known for this alga, the Copper Response Regulator 1 (CRR1) transcription factor is responsible for differential transcription of *HYDA1* and *Ferredoxin 5* (*FDX5*) under copper (Cu) deficiency and hypoxia [189–193]. Transcript levels encoding for typical anaerobic proteins, such as *HYDG*, *HYDEF* and *PFR1*, also increase in response to Cu limitation [194], suggesting the involvement of common regulatory pathways.

CRR1 binds the GTAC motif which is the core of Cu response elements (CuREs) [189,195], and two GTAC motifs have also been identified in *HYDA1*[193]. In spite of the overlap between Cuand oxygen-deficiency responses, promoter studies suggest that additional factors specific for each environmental condition influence *HYDA1* transcription, by tuning protein levels to metabolic needs [193]. The identification of these unknown factor(s) is currently a hot topic in *Chlamydomonas* research and will help us in understanding how the fermentative metabolism is modulated and restructured in response to different environmental conditions.

## 5. Concluding Remarks

Global climate change has a deep impact on the frequency and magnitude of precipitations. This causes severe events such as drought and floods [2] that have a dramatically negative impact on crop production. As a consequence of these changes, contrasting environmental conditions, once limited to different climatic areas, can now often occur sequentially in the same geographic area and during a single crop cycle. The impact of flooding on crops is dramatic and causes huge economic losses worldwide [2]. Even crops that are relatively flood-tolerant, such as rice, suffer from prolonged flooding. The recent discovery of SUB1A as the gene able to confer enhanced tolerance to submergence in rice resulted in the development of new rice varieties that display an exceptional tolerance to submergence coupled to high yield [2]. Crops different from rice are usually extremely flood-intolerant. The recent discovery of the oxygen sensing mechanism in plants [41,42] opens new perspectives for the development of crop varieties that can withstand floods. In the context of the responses of algae to low oxygen, the importance of *Chlamydomonas* as hydrogen-producing organisms makes the studies of this organism important, because *Chlamydomonas* are able to produce hydrogen only under anoxia [153]. The development of *Chlamydomonas* strains that can better tolerate anoxia would contribute to the efficiency of hydrogen production by this organism. These examples highlight the importance of research on plant and algae responses to low-oxygen. Several aspects of the molecular responses to hypoxia are still incomplete. These include the role of reactive oxygen species, the possible cross-talk and partially overlapping signaling pathways among different abiotic stresses, and, most importantly, the development of a set of new plant varieties that can guarantee elevated yields even under critical environmental conditions such as those resulting from waterlogging and flooding.

## References

[1] Drew M.C. (1997). Oxygen deficiency and root metabolism: Injury and acclimation under hypoxia and anoxia. Annu. Rev. Plant Physiol. Plant Mol. Biol.

[2] Bailey-Serres J., Fukao T., Gibbs D.J., Holdsworth M.J., Lee S.C., Licausi F., Perata P., Voesenek L.A.C.J., van Dongen J.T. (2012). Making sense of low oxygen sensing. Trends Plant Sci.

[3] Gupta K.J., Zabalza A., van Dongen J.T. (2009). Regulation of respiration when the oxygen availability changes. Physiol. Plantarum.

[4] Zabalza A., van Dongen J.T., Froehlich A., Oliver S.N., Faix B., Gupta K.J., Schmalzlin E., Igal M., Orcaray L., Royuela M. (2009). Regulation of respiration and fermentation to control the plant internal oxygen concentration. Plant Physiol.

[5] Kennedy R.A., Rumpho M.E., Fox T.C. (1992). Anaerobic metabolism in plants. Plant Physiol.

[6] Ricard B., Couee I., Raymond P., Saglio P.H., Saint-Ges V., Pradet A. (1994). Plant metabolism under hypoxia and anoxia. Plant Physiol. Biochem.

[7] Gibbs J., Morrell S., Valdez A., Setter T.L., Greenway H. (2000). Regulation of alcoholic fermentation in coleoptiles of two rice cultivars differing in tolerance to anoxia. J. Exp. Bot.

[8] Geigenberger P. (2003). Response of plant metabolism to too little oxygen. Curr. Opin. Plant Biol.

[9] Rivoal J., Ricard B., Pradet A. (1991). Lactate dehydrogenase in *Oryza sativa* L. seedlings and roots: Identification and partial characterization. Plant Physiol.

[10] Rivoal J., Hanson A. (1994). Metabolic control of anaerobic glycolysis: Overexpression of lactate dehydrogenase in transgenic tomato roots supports the Davies-Roberts hypothesis and points to a critical role for lactate secretion. Plant Physiol.

[11] Christopher M.E., Good A.G. (1996). Characterization of hypoxically inducible lactate dehydrogenase in maize. Plant Physiol.

[12] Sweetlove L.J., Dunford R., Ratcliffe R.G., Kruger N.J. (2000). Lactate metabolism in potato tubers deficient in lactate dehydrogenase activity. Plant Cell Environ.

[13] Roberts J.K., Callis J., Jardetzky O., Walbot V., Freeling M. (1984). Cytoplasmic acidosis as a determinant of flooding intolerance in plants. Proc. Natl. Acad. Sci. USA.

[14] Felle H.H. (2005). pH regulation in anoxic plants. Ann. Bot.

[15] Licausi F., Perata P. (2009). Low oxygen signaling and tolerance in plants. Adv. Bot. Res.

[16] Choi W.G., Roberts D.M. (2007). *Arabidopsis* NIP2;1, a major intrinsic protein transporter of lactic acid induced by anoxic stress. J. Biol. Chem.

[17] Dolferus R., Wolansky M., Carroll R.R., Miyashita R.Y., Ismond K.K., Good A. (2008). Functional analysis of lactate dehydrogenase during hypoxic stress in *Arabidopsis*. Funct. Plant Biol.

[18] Perata P., Alpi A. (1993). Plant responses to anaerobiosis. Plant Sci.

[19] Dolferus R., Ellis M., de Bruxelles G., Trevaskis B., Hoeren F., Dennis E.S., Peacock W.J. (1997). Strategies of gene action in *Arabidopsis* during hypoxia. Ann. Bot.

[20] Ellis M.H., Dennis E.S., Peacock W.J. (1999). *Arabidopsis* roots and shoots have different mechanisms for hypoxic stress tolerance. Plant Physiol.

[21] Dolferus R., Klok E.J., Delessert C., Wilson S., Ismond K.P., Good A.G., Peacock W.J., Dennis E.S. (2003). Enhancing the anaerobic response. Ann. Bot.

[22] Visser E.J.W., Voesenek L.A.C.J., Vartapetian B.B., Jackson M.B. (2003). Flooding and plant growth. Ann. Bot.

[23] Loreti E., Poggi A., Novi G., Alpi A., Perata P. (2005). A genome-wide analysis of the effects of sucrose on gene expression in *Arabidopsis* seedlings under anoxia. Plant Physiol.

[24] Bucher M., Brädle R., Khuhlemeier C. (1994). Ethanolic fermentation in transgenic tobacco expressing *Zymomonas mobilis* pyruvate decarboxylase. EMBO J.

[25] Kürsteiner O., Dupuis I., Kuhlemeier C. (2003). The *pyruvate decarboxylase1* gene of *Arabidopsis* is required during anoxia but not other environmental stresses. Plant Physiol.

[26] Mithran M., Paparelli E., Novi G., Perata P., Loreti E (2013). Analysis of the role of the pyruvate decarboxylase gene family in *Arabidopsis thaliana* under low oxygen conditions. Plant Biol..

[27] Ismond K.P., Dolferus R., de Pauw M., Dennis E.S., Good A.G. (2003). Enhanced low oxygen survival in *Arabidopsis* through increased metabolic flux in the fermentative pathway. Plant Physiol.

[28] Jacobs M., Dolferus R., van Den Bosshe V.B. (1988). Isolation and biochemical analysis of ethyl methyl sulfonate induced alcohol dehydrogenase null mutants of *Arabidopsis thaliana* (L.) Heynh. Biochem. Genet.

[29] Matsumura H., Takano T., Yoshida K.T., Takeda G. (1995). A rice mutant lacking alcohol dehydorogenase. Breeding Sci.

[30] Matsumura H., Takano T., Takeda G., Uchimiya H. (1998). *Adh1* is transcriptionally active but its translational product is reduced in a *rad* mutant of rice (*Oryza sativa* L.), which is vulnerable to submergence stress. Theor. Appl. Genet.

[31] Johnson J.R., Cobb B.G., Drew M.C. (1994). Hypoxic induction of anoxia tolerance in roots of Adh null *Zea mays*. Plant Physiol.

[32] Shiao T., Ellis M.H., Dolferus R., Dennis E.S., Doran P.M. (2002). Overexpression of alcohol dehydrogenase or pyruvate decarboxylase improves the growth of hairy roots under hypoxia. Biotechnol. Bioeng.

[33] Magneschi L., Perata P. (2009). Rice germination and seedling growth in the absence of oxygen. Ann. Bot.

[34] Rocha M., Licausi F., Araújo W.L., Nunes-Nesi A., Sodek L., Fernie A.R., van Dongen J.T. (2010). Glycolysis and the tricarboxylic acid cycle are linked by alanine aminotransferase during hypoxia induced by waterlogging of *Lotus japonicus*. Plant Physiol.

[35] Branco-Price C., Kaiser K.A., Jang C.J., Larive C.K., Bailey-Serres J. (2008). Selective mRNA translation coordinates energetic and metabolic adjustments to cellular oxygen deprivation and reoxygenation in *Arabidopsis thaliana*. Plant J.

[36] Ricoult C., Cliquet J.B., Limami A.M. (2005). Stimulation of alanine amino transferase (AlaAT) gene expression and alanine accumulation in embryo axis of the model legume *Medicago truncatula* contribute to anoxia stress tolerance. Physiol. Plantarum.

[37] Miyashita Y., Dolferus R., Ismond K.P., Good A.G. (2007). Alanine aminotransferase catalyses the breakdown of alanine after hypoxia in *Arabidopsis thaliana*. Plant J.

[38] Aurisano N., Bertani A., Reggiani R. (1995). Involvement of calcium and calmodulin in protein and amino acid metabolism in rice roots under anoxia. Plant Cell Physiol.

[39] Breitkreuz K.E., Allan W.L., van Cauwenberghe O.R., Jakobs C., Talibi D., Andre B., Shelp B.J. (2003). Anovel γ-hydroxybutyrate dehydrogenase: Identification and expression of an *Arabidopsis* cDNA and potential role under oxygen deficiency. J. Biol. Chem.

[40] Sweetlove L.J., Beard K.F.M., Nunes-Nesi A., Fernie A.R., Ratcliffe R.G. (2010). Not just a circle: Flux modes in the plant TCA cycle. Trends Plant Sci.

[41] Gibbs D.J., Lee S.C., Isa N.M., Gramuglia S., Fukao T., Bassel G.W., Correia C.S., Corbineau F., Theodoulou F.L., Bailey-Serres J. (2011). Homeostatic response to hypoxia is regulated by the *N*-end rule pathway in plants. Nature.

[42] Licausi F., Kosmacz M., Weits D.A., Giuntoli B., Giorgi F.M., Voesenek L.A.C.J., Perata P., van Dongen J.T. (2011). Oxygen sensing in plants is mediated by an *N*-end rule pathway for protein destabilization. Nature.

[43] Roitsch T. (1999). Source-sink regulation by sugar and stress. Curr. Opin. Plant Biol.

[44] Blasing O.E., Gibon Y., Gunther M., Hohne M., Morcuende R. (2005). Sugars and circadian regulation make major contributions to the global regulation of diurnal gene expression in Arabidopsis. Plant Cell.

[45] Xia J.H., Saglio P.H. (1992). Lactic Acid efflux as a mechanism of hypoxic acclimation of maize root tips to anoxia. Plant Physiol.

[46] Sairam R.K., Dharmar K., Chinnusamy V., Meena R.C. (2009). Waterlogging-induced increase in sugar mobilization, fermentation, and related gene expression in the roots of mung bean (*Vigna radiata*). J. Plant Physiol.

[47] Vartapetian B.B., Jackson M.B. (1997). Plant adaptation to anaerobic stress. Ann. Bot.

[48] Sturm A., Hess D., Lee H.S., Lienhard S. (1999). Neutral invertase is a novel type of sucrose–cleaving enzyme. Physiol. Plant.

[49] Bailey-Serres J., Voesenek L.A.C.J. (2008). Flooding stress: Acclimations and genetic diversity. Annu. Rev. Plant Biol.

[50] Barclay A.M., Crawford R.M. (1983). The effect of anaerobiosis on carbohydrate levels in storage tissues of wetland plants. Ann. Bot.

[51] Bertrand A., Castonguay Y., Nadeau P., Laberge S., Michaud R., Bélanger G., Rochette P. (2003). Oxygen deficiency affects carbohydrate reserves in overwintering forage crops. J. Exp. Bot.

[52] Stitt M. (1990). Fructose-2,6-bisphosphate as a regulatory molecule in plants. Trends Plant Sci.

[53] Smith A.M., Zeeman S.C., Smith S.M. (2005). Starch degradation. Ann. Rev. Plant Biol.

[54] Germain V., Raymond P., Ricard B. (1997). Differential expression of two tomato lactate dehydrogenase genes in response to oxygen deficit. Plant Mol. Biol.

[55] Zeng Y., Wu Y., Avigne W.T., Koch K.E. (1999). Rapid repression of maize invertases by low oxygen. Invertase/sucrose synthase balance, sugar signaling potential, and seedling survival. Plant Physiol.

[56] Van Dongen J.T., Roeb G.W., Dautzenberg M., Froehlich A., Vigeolas H., Minchin P.E.H., Geigenberger P. (2004). Phloem import and storage metabolism are highly coordinated by the low oxygen concentrations within developing wheat seeds. Plant Physiol.

[57] Mustroph A., Albrecht G., Hajirezaei M., Grimm B., Biemelt S. (2005). Low levels of pyrophosphate in transgenic potato plants expressing *E. coli* pyrophosphatase lead to decreased vitality under oxygen deficiency. Ann. Bot.

[58] Zeng Y., Wu Y., Avigne W.T., Koch K.E. (1998). Differential regulation of sugar-sensitive sucrose synthases by hypoxia and anoxia indicate complementary transcriptional and posttranscriptional responses. Plant Physiol.

[59] Baud S., Vaultier M.N., Rochat C. (2004). Structure and expression profile of the sucrose synthase multigene family in *Arabidopsis*. J. Exp. Bot.

[60] Bieniawska Z., Paul Barratt D.H., Garlick A.P., Thole V., Kruger N.J. (2007). Analysis of the sucrose synthase gene family in *Arabidopsis*. Plant J.

[61] Guglielminetti L., Morita A., Yamaguchi J., Loreti E., Perata P., Alpi A. (2006). Differential expression of two fructokinases in *Oryza sativa* seedlings grown under aerobic and anaerobic conditions. J. Plant Res.

[62] Cho J.I., Ryoo N., Ko S., Lee S.K., Lee J., Jung K.H., Lee Y.H., Bhoo S., Winderickx J., An G. (2006). Structure, expression, and functional analysis of the hexokinase gene family in rice (*Oryza sativa* L.). Planta.

[63] Lasanthi-Kudahettige R., Magneschi L., Loreti E., Gonzali S., Licausi F., Novi G., Beretta O., Vitulli F., Alpi A., Perata P. (2007). Transcript profiling of the anoxic rice coleoptile. Plant Physiol.

[64] Mertens E., Larondelle Y., Hers H.G. (1990). Induction of pyrophosphate: Fructose 6-phosphate 1-phosphotransferase by anoxia in rice seedlings. Plant Physiol.

[65] Mohanty B., Wilson P.M., ap Rees T. (1993). Effects of anoxia on growth and carbohydrate metabolism in suspension cultures of soybean and rice. Phytochemistry.

[66] Faiz-Ur-Rahman A.T.M., Trewavas A.J., Davies D.D. (1974). The Pasteur effect in carrot root tissue. Planta.

[67] Moons A., Valcke R., van Montagu M. (1998). Low-oxygen stress and water deficit induce cytosolic pyruvate orthophosphate dikinase (PPDK) expression in roots of rice, a C3 plant. Plant J.

[68] Kaelin W.G., Ratcliffe P.J. (2008). Oxygen sensing by metazoans: The central role of the HIF hydroxylase pathway. Mol. Cell.

[69] Loenarz C., Coleman M.L., Boleininger A., Schierwater B., Holland P.W.H., Ratcliffe P.J., Schofield C.J. (2011). The hypoxia-inducible transcription factor pathway regulates oxygen sensing in the simplest animal, *Trichoplax. adhaerens*. EMBO Rep.

[70] Sasidharan R., Mustroph A. (2011). Plant oxygen sensing is mediated by the *N*-end rule pathway: A milestone in plant anaerobiosis. Plant Cell.

[71] Mus F., Dubini A., Seibert M., Posewitz M.C., Grossman A.R. (2007). Anaerobic acclimation in *Chlamydomonas reinhardtii*, anoxic gene expression, hydrogenase induction, and metabolic pathways. J. Biol. Chem.

[72] Bachmair A., Finley D., Varshavsky A. (1986). *In vivo* half-life of a protein is a function of its amino-terminal residue. Science.

[73] Varshavsky A. (2011). The *N*-end rule pathway and regulation by proteolysis. Protein Sci.

[74] Prakash S., Tian L., Ratliff K.S., Lehotzky R.E., Matouschek A. (2004). An unstructured initiation site is required for efficient proteasome-mediated degradation. Nat. Struct. Mol. Biol.

[75] Ditzel M., Wilson R., Tenev T., Zachariou A., Paul A., Deas E., Meier P. (2003). Degradation of DIAP1 by the *N*-end rule pathway is essential for regulating apoptosis. Nat. Cell Biol.

[76] Mogk A., Schmidt R., Bukau B. (2007). The *N*-end rule pathway for regulated proteolysis: Prokaryotic and eukaryotic strategies. Trends Cell Biol.

[77] Graciet E., Mesiti F., Wellmer F. (2010). Structure and evolutionary conservation of the plant *N*-end rule pathway. Plant J.

[78] Tasaki T., Kwon Y.T. (2007). The mammalian *N*-end rule pathway: New insights into its components and physiological roles. Trends Biochem. Sci.

[79] Graciet E., Wellmer F. (2010). The plant *N*-end rule pathway: Structure and functions. Trends Plant Sci.

[80] Yoshida S., Ito M., Callis J., Nishida I., Watanabe A. (2002). A delayed leaf senescence mutant is defective in arginyl-tRNA: Proteinarginyltransferase, a component of the *N*-end rule pathway in Arabidopsis. Plant J.

[81] Graciet E., Walter F., Maoileidigh D.O., Pollmann S., Meyerowitz E.M., Varshavsky A., Wellmer F. (2009). The *N*-end rule pathway controls multiple functions during *Arabidopsis* shoot and leaf development. Proc. Natl. Acad. Sci. USA.

[82] Holman T.J., Jones P.D., Russell L., Medhurst A., Ubeda T.S., Talloji P., Marquez J., Schmuths H., Tung S.A., Taylor I. (2009). The *N*-end rule pathway promotes seed germination and establishment through removal of ABA sensitivity in *Arabidopsis*. Proc. Natl. Acad. Sci. USA.

[83] Nakano T., Suzuki K., Fujimura T., Shinshi H. (2006). Genome-wide analysis of the *ERF* gene family in *Arabidopsis* and rice. Plant Physiol.

[84] Xu K., Xu X., Fukao T., Canlas P., Maghirang-Rodriguez R., Heuer S., Ismail A.M., Bailey-Serres J., Ronald P.C., Mackill D.J. (2006). Sub1A is an ethylene-response-factor-like gene that confers submergence tolerance to rice. Nature.

[85] Papdi C., Abraham E., Joseph M.P., Popescu C., Koncz C., Szabados L. (2008). Functional identification of *Arabidopsis* stress regulatory genes using the controlled cDNA overexpression system. Plant Physiol..

[86] Hattori Y., Nagai K., Furukawa S., Song X.-J., Kawano R., Sakakibara H., Wu J., Matsumoto T., Yoshimura A., Kitano H. (2009). The ethylene response factors SNORKEL1 and SNORKEL2 allow rice to adapt to deep water. Nature.

[87] Licausi F., van Dongen J.T., Giuntoli B., Novi G., Santaniello A., Geigenberger P., Perata P. (2010). HRE1 and HRE2, two hypoxia-inducible ethylene response factors, affect anaerobic responses in *Arabidopsis thaliana*. Plant J.

[88] Hu R.-G., Sheng J., Qi X., Xu Z., Takahashi T.T., Varshavsky A. (2005). The *N*-end rule pathway as a nitric oxide sensor controlling the levels of multiple regulators. Nature.

[89] Mustroph A., Lee S.C., Oosumi T., Zanetti M.E., Yang H., Ma K., Yaghoubi-Masihi A., Fukao T., Bailey-Serres J. (2010). Cross-kingdom comparison of transcriptomic adjustments to low oxygen stress highlights conserved and plant-specific responses. Plant Physiol.

[90] Pucciariello C., Parlanti S., Banti V., Novi G., Perata P. (2012). Reactive oxygen species-driven transcription in *Arabidopsis* under oxygen deprivation. Plant Physiol.

[91] Vandenbroucke K., Robbens S., Vandepoele K., Inzè D., van de Peer Y., van Breusegem F. (2008). Hydrogen peroxide-induced gene expression across kingdoms: A comparative analysis. Mol. Biol. Evol.

[92] Banti V., Mafessoni F., Loreti E., Alpi A., Perata P. (2010). The heat-inducible transcription factor *HsfA2* enhances anoxia tolerance in *Arabidopsis*. Plant Physiol.

[93] Mittler R., Vanderauwera S., Gollery M., van Breusegem F. (2004). Reactive oxygen gene network of plants. Trends Plant Sci.

[94] Davletova S., Schlauch K., Coutu J., Mittler R. (2005). The zinc-finger protein Zat12 plays a central role in reactive oxygen and abiotic stress signaling in *Arabidopsis*. Plant Physiol.

[95] Baxter-Burrell A., Yang Z., Springer P.S., Bailey-Serres J. (2002). RopGAP4-dependent Rop GTPase rheostat control of *Arabidopsis* oxygen deprivation tolerance. Science.

[96] Chang R., Jang C.J.H., Branco-Price C., Nghiem P., Bailey-Serres J. (2012). Transient MPK6 activation in response to oxygen deprivation and reoxygenation is mediated by mitochondria and aids seedling survival in *Arabidopsis*. Plant Mol. Biol.

[97] Pucciariello C., Perata P. (2012). How plants sense low oxygen. Plant Signal. Behav.

[98] Licausi F., Pucciariello C., Perata P. (2012). New role for an old rule: *N*-end rule mediated degradation of ERF proteins governs low oxygen response in plants. J. Integr. Plant Biol.

[99] Miki H., Funato Y. (2012). Regulation of intracellular signalling through cysteine oxidation by reactive oxygen species. J. Biochem.

[100] Rhee S.G., Chae H.Z., Kim K. (2005). Peroxiredoxins: A historical overview and speculative preview of novel mechanisms and emerging concepts in cell signaling. Free Radic. Biol. Med.

[101] Gupta R., Luan S. (2003). Redox control of protein tyrosine phosphatases and mitogen-activated protein kinases in plants. Plant Physiol.

[102] Blanchetot C., Tertoolen L.G.J., Hertog J.D. (2002). Regulation of receptor protein-tyrosine phosphatase a by oxidative stress. EMBO J.

[103] Igamberdiev A.U., Bykova N.V., Shah J.K., Hill R.D. (2010). Anoxic nitric oxide cycling in plants: Participating reactions and possible mechanisms. Physiol. Plantarum.

[104] Gupta K.J., Igamberdiev A.U., Manjunatha G., Segu S., Moran J.F., Neelawarne B., Bauwe H., Kaiser W.M. (2011). The emerging roles of nitric oxide (NO) in plant mitochondria. Plant Sci.

[105] Li F., Sonveaux P., Rabbani Z.N., Liu S., Yan B., Huang Q., Vujaskovic Z., Dewhirst M.W., Li C.Y. (2007). Regulation of HIF-1 alpha stability through *S*-nitrosylation. Mol. Cell.

[106] Ho J.J.D., Man H.S.J., Marsden P.A. (2012). Nitric oxide signaling in hypoxia. J. Mol. Med.

[107] Banti V., Loreti E., Novi G., Santaniello A., Alpi A., Perata P. (2008). Heat acclimation and cross tolerance against anoxia in Arabidopsis. Plant Cell Environ.

[108] Kim H.S., Snesrud E.C., Moy L.P., Haas B.J., Nierman W.C., Quackenbush J. (2005). Transcriptional divergence of the duplicated oxidative stress-responsive genes in the *Arabidopsis* genome. Plant J.

[109] Gadjev I., Vanderauwera S., Gechev T.S., Laloi C., Minkov I.N., Shulaev V., Apel K., Inzè D., Mittler R., van Breusegem F. (2006). Transcriptomic footprints disclose specificity of reactive oxygen species signalling in *Arabidopsis*. Plant Physiol.

[110] Van Dongen J.T., Fröhlic A., Ramìrez-Aguilar S.J., Schauer N., Fernie A.R., Erban A., Kopka J., Clark J., Langer A., Geigenberger P. (2008). Transcript and metabolic profiling of the adaptive response to mild decreases in oxygen concentration in the roots of *Arabidopsis* plants. Ann. Bot. Lond.

[111] Vierling E. (1991). The role of heat shock proteins in plants. Ann. Rev. Plant Physiol.

[112] Nishizawa A., Yabuta Y., Yoshida E., Maruta T., Yoshimura K., Shigeoka S. (2006). *Arabidopsis* heat shock transcription factor A2 as a key regulator in response to several types of environmental stress. Plant J.

[113] Schramm F., Ganguli A., Kiehlmann E., Englich G., Walch D., von Koskull-Döring P. (2006). The heat stress transcription factor *HsfA2* serves as a regulatory amplifier of a subset of genes in the heat stress response of *Arabidopsis*. Plant Mol. Biol.

[114] Ogawa D., Yamaguchi K., Nishiuchi T. (2007). High-level overexpression of the *Arabidopsis HsfA2* gene confers not only increased themotolerance but also salt/osmotic stress tolerance and enhanced callus growth. J. Exp. Bot.

[115] Nishizawa-Yokoi A., Tainaka H., Yoshida E., Tamoi M., Yabuta Y., Shigeoka S. (2010). The 26S proteasome function and Hsp90 activity involved in the regulation of *HsfA2* expression in response to oxidative stress. Plant Cell Physiol.

[116] Nishizawa A., Nosaka Y.R., Hayashi H., Tainaka H., Maruta T., Tamoi M., Ikeda M., Takagi M.O., Yoshimura K., Yabuta Y. (2011). *HsfA1d* and *HsfA1e* involved in the transcriptional regulation of *HsfA2* function as key regulators for the Hsf signaling network in response to environmental stress. Plant Cell Physiol.

[117] Yu H.D., Yang X.F., Chen S.T., Wang Y.T., Li J.K., Shen Q., Liu X.L., Guo F.Q. (2012). Downregulation of chloroplast RPS1 negatively modulates nuclear heat-responsive expression of HsfA2 and its target genes in *Arabidopsis*. PLoS Genet.

[118] Zhang L., Li Y., Xing D., Gao C. (2009). Characterization of mitochondrial dynamics and subcellular localization of ROS reveal that HsfA2 alleviates oxidative damage caused by heat stress in Arabidopsis. J. Exp. Bot.

[119] Bissoli G., Niñoles R., Fresquet S., Palombieri S., Bueso E., Rubio L., García-Sánchez M.J., Fernández J.A., Mulet J.M., Serrano R. (2012). Peptidyl-prolyl *cis*-*trans* isomerase ROF2 modulates intracellular pH homeostasis in *Arabidopsis*. Plant J.

[120] Pucciariello C., Banti V., Perata P. (2012). ROS signaling as common element in low oxygen and heat stresses. Plant Physiol. Bioch.

[121] Blokhina O.B., Chirkova T.V., Fagerstedt K.V. (2001). Anoxic stress leads to hydrogen peroxide formation in plant cells. J. Exp. Bot.

[122] Fukao T., Bailey-Serres J. (2004). Plant responses to hypoxia—Is survival a balancing act?. Trends Plant Sci.

[123] Tsuji H. (1973). Growth and metabolism in plants under anaerobic conditions. Environ. Control. Biol.

[124] Huang S.B., Greenway H., Colmer T.D. (2003). Anoxia tolerance in rice seedlings: Exogenous glucose improves growth of an anoxia-“intolerant”, but not of a “tolerant” genotype. J. Exp. Bot.

[125] Murata T., Akazawa T., Fukuchi S. (1968). Enzymic mechanism of starch breakdown in germinating rice seeds: 1. An analytical study. Plant Physiol.

[126] Dunn G. (1974). A model for starch breakdown in higher plants. Phytochemistry.

[127] Sun Z., Henson C.A. (1991). A quantitative assessment of the importance of barley seed α-amylase, debranching enzyme, and α-glucosidase in starch degradation. Arch. Biochem. Biophys.

[128] Perata P., Pozuetaromero J., Akazawa T., Yamaguchi J. (1992). Effect of anoxia on starch breakdown in rice and wheat seeds. Planta.

[129] Guglielminetti L., Yamaguchi J., Perata P., Alpi A. (1995). Amylolytic activities in cereal seeds under aerobic and anaerobic conditions. Plant Physiol.

[130] Perata P., Matsukura C., Vernieri P., Yamaguchi J. (1997). Sugar repression of a gibberellin-dependent signaling pathway in barley embryos. Plant Cell.

[131] Morita A., Umemura T., Kuroyanagi M., Futsuhara Y., Perata P., Yamaguchi J. (1998). Functional dissection of a sugar-repressed α-amylase gene (*RAmy1A*) promoter in rice embryos. FEBS Lett.

[132] Loreti E., Yamaguchi J., Alpi A., Perata P. (2003). Gibberellins are not required for rice germination under anoxia. Plant Soil.

[133] Lu C.A., Ho T.H.D., Ho S.L., Yu S.M. (2002). Three novel MYB proteins with one DNA binding repeat mediate sugar and hormone regulation of α-amylase gene expression. Plant Cell.

[134] Lu C.-A., Lin C.-C., Lee K.-W., Chen J.-L., Huang L.-F., Ho S.-L., Liu H.-J., Hsing Y.-I., Yu S.-M. (2007). The SnRK1A protein kinase plays a key role in sugar signaling during germination and seedling growth of rice. Plant Cell.

[135] Lee K.-W., Chen P.-W., Lu C.-A., Chen S., Ho T.-H.D., Yu S.-M. (2009). Coordinated responses to oxygen and sugar deficiency allow rice seedlings to tolerate flooding. Sci. Signal..

[136] Kudahettige N.P., Pucciariello C., Parlanti S., Alpi A., Perata P. (2011). Regulatory interplay of the Sub1A and CIPK15 pathways in the regulation of α-amylase production in flooded rice plants. Plant Biol.

[137] Lu C.-A., Lim E.-K., Yu S.-M. (1998). Sugar response sequence in the promoter of a rice α-amylase gene serves as a transcriptional enhancer. J. Biol. Chem.

[138] Toyofuku K., Umemura T, Yamaguchi J. (1998). Promoter elements required for sugar-repression of the *RAmy3D* gene for α-amylase in rice. FEBS Lett..

[139] Chen P.-W., Lu C.-A., Yu T.-S., Tseng T.-H., Wang C.-S., Yu S.-M. (2002). Rice α-amylase transcriptional enhancers direct multiple mode regulation of promoters in transgenic rice. J. Biol. Chem.

[140] Cosgrove D.J. (1999). Enzymes and other agents that enhance cell wall extensibility. Annu. Rev. Plant Physiol. Plant Mol. Biol.

[141] Huang J., Takano T., Akita S. (2000). Expression of α-expansin genes in young seedlings of rice (*Oryza sativa* L.). Planta.

[142] Magneschi L., Kudahettige R.L., Alpi A., Perata P. (2009). Expansin gene expression and anoxic coleoptile elongation in rice cultivars. J. Plant Physiol.

[143] Choi D.S., Lee Y., Cho H.T., Kende H. (2003). Regulation of expansin gene expression affects growth and development in transgenic rice plants. Plant Cell.

[144] Li Z.-X., Septiningsih E.M., Quilloy-Mercado S.M., McNally K.L., Mackill D.J. (2011). Identification of *SUB1A* alleles from wild rice *Oryza rufipogon* Griff. Genet. Resour. Crop. Ev.

[145] Niroula R.K., Pucciariello C., Ho V.T., Novi G., Fukao T., Perata P. (2012). SUB1A-dependent and -independent mechanisms are involved in the flooding tolerance of wild rice species. Plant J.

[146] Vergara B.S., Mazaredo A (1975). Screening for resistance to submergence under greenhouse conditions. Proceedings of the International Seminar on Deepwater Rice.

[147] Nagai K., Hattori Y., Ashikari M. (2010). Stunt or elongate? Two opposite strategies by which rice adapts to floods. J. Plant Res.

[148] Xu K., Mackill D.J. (1996). A major locus for submergence tolerance mapped on rice chromosome 9. Mol. Breed.

[149] Fukao T., Xu K., Ronald P.C., Bailey-Serres J. (2006). A variable cluster of ethylene response factor- like genes regulates metabolic and developmental acclimation responses to submergence in rice. Plant Cell.

[150] Fukao T., Bailey-Serres J. (2008). Submergence tolerance conferred by *Sub1A* is mediated by SLR1 and SLRL1 restriction of gibberellin responses in rice. Proc. Natl. Acad. Sci. USA.

[151] Septiningsih E.M., Pamplona A.M., Sanchez D.L., Neeraja C.N., Vergara G.V., Heuer S., Ismail A.M., Mackill D.J. (2009). Development of submergence-tolerant rice cultivars: The Sub1 locus and beyond. Ann. Bot.

[152] Singh N., Dang T., Vergara G., Pandey D., Sanchez D., Neeraja C., Septiningsih E., Mendioro M., Tecson-Mendoza R., Ismal A. (2010). Molecular marker survey and expression analyses of the rice submergence-tolerance genes *SUB1A* and *SUB1C*. Theor. Appl. Genet.

[153] Grossman A.R., Catalanotti C., Yang W., Dubini A., Magneschi L., Subramanian V., Posewitz M.C., Seibert M. (2011). Multiple facets of anoxic metabolism and hydrogen production in the unicellular green alga *Chlamydomonas reinhardtii*. New Phytol.

[154] Merchant S.S., Prochnik S.E., Vallon O., Harris E.H., Karpowicz S.J., Witman G.B., Terry A., Salamov A., Fritz-Laylin L.K., Maréchal-Drouard L. (2007). The *Chlamydomonas* genome reveals the evolution of key animal and plant functions. Science.

[155] Harris E.H. (2001). *Chlamydomonas* as a model organism. Annu. Rev. Plant Physiol. Plant Mol. Biol.

[156] Grossman A.R. (2007). In the grip of algal genomics. Adv. Exp. Med. Biol.

[157] Purton S. (2007). Tools and techniques for chloroplast transformation of *Chlamydomonas Adv*. Exp. Med. Biol.

[158] Molnar A., Bassett A., Thuenemann E., Schwach F., Karkare S., Ossowski S., Weigel D., Baulcombe D. (2009). Highly specific gene silencing by artificial microRNAs in the unicellular alga *Chlamydomonas reinhardtii*. Plant J.

[159] Gonzalez-Ballester D., Pootakham W., Mus F., Yang W., Catalanotti C., Magneschi L., de Montaigu A., Higuera J.J., Prior M., Galván A. (2011). Reverse genetics in *Chlamydomonas*, a platform for isolating insertional mutants. Plant Methods.

[160] Pootakham W., Gonzalez-Ballester D., Grossman A.R. (2010). Identification and regulation of plasma membrane sulfate transporters in *Chlamydomonas*. Plant Physiol.

[161] Schmollinger S., Strenkert D., Schroda M. (2010). An inducible artificial microRNA system for *Chlamydomonas reinhardtii* confirms a key role for heat shock factor 1 in regulating thermotolerance. Curr. Genet.

[162] Godman J.E., Molnár A., Baulcombe D.C., Balk J. (2010). RNA silencing of hydrogenase(-like) genes and investigation of their physiological roles in the green alga *Chlamydomonas reinhardtii*. Biochem. J.

[163] Meuser J.E., D’Adamo S., Jinkerson R.E., Mus F., Yang W., Ghirardi M.L., Seibert M., Grossman A.R., Posewitz M.C. (2012). Genetic disruption of both *Chlamydomonas reinhardtii* [FeFe]-hydrogenases, insight into the role of HYDA2 in H_2_ production. Biochem. Biophys. Res. Commun.

[164] Forestier M., King P., Zhang L., Posewitz M., Schwarzer S., Happe T., Ghirardi M.L., Seibert M. (2003). Expression of two [Fe]-hydrogenases in *Chlamydomonas reinhardtii* under anaerobic conditions. Eur. J. Biochem.

[165] Happe T., Kaminski A. (2002). Differential regulation of the Fe-hydrogenase during anaerobic adaptation in the green alga *Chlamydomonas reinhardtii*. Eur. J. Biochem.

[166] Posewitz M.C., King P.W., Smolinski S.L., Zhang L., Seibert M., Ghirardi M.L. (2004). Discovery of two novel radical *S*-adenosylmethionine proteins required for the assembly of an active [Fe]-hydrogenase. J. Biol. Chem.

[167] Posewitz M.C., King P.W., Smolinski S.L., Smith R.D., Ginley A.R., Ghirardi M.L., Seibert M. (2005). Identification of genes required for hydrogenase activity in *Chlamydomonas reinhardtii*. Biochem. Soc. Trans.

[168] Atteia A., van Lis R., Gelius-Dietrich G., Adrait A., Garin J., Joyard J., Rolland N., Martin W. (2006). Pyruvate formate-lyase and a novel route of eukaryotic ATP synthesis in *Chlamydomonas mitochondria*. J. Biol. Chem.

[169] Terashima M., Specht M., Naumann B., Hippler M. (2010). Characterizing the anaerobic response of *Chlamydomonas reinhardtii* by quantitative proteomics. Mol. Cell Proteomics.

[170] Philipps G., Krawietz D., Hemschemeier A., Happe T. (2011). A pyruvate formate lyase-deficient *Chlamydomonas reinhardtii* strain provides evidence for a link between fermentation and hydrogen production in green algae. Plant J.

[171] Terashima M., Specht M., Hippler M. (2011). The chloroplast proteome, a survey from the *Chlamydomonas reinhardtii* perspective with a focus on distinctive features. Curr. Genet.

[172] Catalanotti C., Dubini A., Subramanian V., Yang W., Magneschi L., Mus F., Seibert M., Posewitz M.C., Grossman A.R. (2012). Altered fermentative metabolism in *Chlamydomonas reinhardtii* mutants lacking pyruvate formate lyase and both pyruvate formate lyase and alcohol dehydrogenase. Plant Cell.

[173] Magneschi L., Catalanotti C., Subramanian V., Dubini A., Yang W., Mus F., Posewitz M.C., Seibert M., Perata P., Grossman A.R. (2012). A mutant in the *ADH1* gene of *Chlamydomonas reinhardtii* elicits metabolic restructuring during anaerobiosis. Plant Physiol.

[174] Gfeller R.P., Gibbs M. (1984). Fermentative metabolism of *Chlamydomonas reinhardtii*, I. Analysis of fermentative products from starch in dark and light. Plant Physiol.

[175] Kreuzberg K. (1984). Starch fermentation via formate producing pathway in *Chlamydomonas reinhardtii; Chlorogonium elongatum* and *Chlorella fusca*. Physiol. Plantarum.

[176] Ohta S., Miyamoto K., Miura Y. (1987). Hydrogen evolution as a consumption mode of reducing equivalents in green algal fermentation. Plant Physiol.

[177] Tsygankov A.A., Kosourov S., Seibert M., Ghirardi M.L. (2002). Hydrogen photoproduction under continuous illumination by sulfur-deprived, synchronous *Chlamydomonas reinhardtii* cultures. Int. J. Hydrog. Energ.

[178] Kosourov S., Seibert M., Ghirardi M.L. (2003). Effects of extracellular pH on the metabolic pathways in sulfur-deprived; H_2_-producing *Chlamydomonas reinhardtii* cultures. Plant Cell Physiol.

[179] Dubini A., Mus F., Seibert M., Grossman A.R., Posewitz M.C. (2009). Flexibility in anaerobic metabolism as revealed in a mutant of *Chlamydomonas reinhardtii* lacking hydrogenase activity. J. Biol. Chem.

[180] Melis A., Zhang L., Forestier M., Ghirardi M.L., Seibert M. (2000). Sustained photobiological hydrogen gas production upon reversible inactivation of oxygen evolution in the green alga *Chlamydomonas reinhardtii*. Plant Physiol.

[181] Tolleter D., Ghysels B., Alric J., Petroutsos D., Tolstygina I., Krawietz D., Happe T., Auroy P., Adriano JM., Beyly A. (2011). Plant Cell.

[182] Terashima M., Petroutsos D., Hüdig M., Tolstygina I., Trompelt K., Gäbelein P., Fufezan C., Kudla J., Weinl S., Finazzi G. (2012). Calcium-dependent regulation of cyclic photosynthetic electron transfer by a CAS, ANR1, and PGRL1 complex. Proc. Natl. Acad. Sci. USA.

[183] Antal T.K., Krendeleva T.E., Laurinavichene T.V., Makarova V.V., Ghirardi M.L., Rubin A.B., Tsygankov A.A., Seibert M. (2003). The dependence of algal H_2_ production on Photosystem II and O_2_ consumption activities in sulfur-deprived *Chlamydomonas reinhardtii* cells. BBA.

[184] Bailey-Serres J., Chang R. (2005). Sensing and signalling in response to oxygen deprivation in plants and other organisms. Ann. Bot.

[185] Guzy R.D., Schumacker P.T. (2006). Oxygen sensing by mitochondria at complex III, the paradox of increased reactive oxygen species during hypoxia. Exp. Physiol.

[186] Bell E.L., Chandel N.S. (2007). Mitochondrial oxygen sensing, regulation of hypoxia-inducible factor by mitochondrial generated reactive oxygen species. Essays Biochem.

[187] Whitney L.A., Loreti E., Alpi A., Perata P. (2011). Alcohol dehydrogenase and hydrogenase transcript fluctuations during a day-night cycle in *Chlamydomonas reinhardtii*, the role of anoxia. New Phytol.

[188] Whitney L.A., Novi G., Perata P., Loreti E (2012). Distinct mechanisms regulating gene expression coexist within the fermentative pathways in *Chlamydomonas reinhardtii*. ScientificWorldJournal.

[189] Quinn J.M., Eriksson M., Moseley J.L., Merchant S. (2002). Oxygen deficiency responsive gene expression in *Chlamydomonas reinhardtii* through a copper-sensing signal transduction pathway. Plant Physiol.

[190] Eriksson M., Moseley J.L., Tottey S., Del Campo J.A., Quinn J., Kim Y., Merchant S. (2004). Genetic dissection of nutritional copper signaling in *Chlamydomonas* distinguishes regulatory and target genes. Genetics.

[191] Kropat J., Tottey S., Birkenbihl R.P., Depège N., Huijser P., Merchant S. (2005). A regulator of nutritional copper signaling in *Chlamydomonas* is an SBP domain protein that recognizes the GTAC core of copper response element. Proc. Natl. Acad. Sci. USA.

[192] Lambertz C., Hemschemeier A., Happe T. (2010). Anaerobic expression of the ferredoxin-encoding *FDX5* gene of *Chlamydomonas reinhardtii* is regulated by the Crr1 transcription factor. Eukaryot. Cell.

[193] Pape M., Lambertz C., Happe T., Hemschemeier A. (2012). Differential expression of the *Chlamydomonas* [FeFe]-hydrogenase-encoding *HYDA1* gene is regulated by the copper response regulator 1. Plant Physiol.

[194] Castruita M., Casero D., Karpowicz S.J., Kropat J., Vieler A., Hsieh S.I., Yan W., Cokus S., Loo J.A., Benning C. (2011). Systems biology approach in *Chlamydomonas* reveals connections between copper nutrition and multiple metabolic steps. Plant Cell.

[195] Quinn J.M., Barraco P., Eriksson M., Merchant S. (2000). Coordinate copper- and oxygen-responsive Cyc6 and Cpx1 expression in *Chlamydomonas* is mediated by the same element. J. Biol. Chem.

